# Dogs and the classic route of Guinea Worm transmission: an evaluation of copepod ingestion

**DOI:** 10.1038/s41598-020-58191-4

**Published:** 2020-01-29

**Authors:** Kayla B. Garrett, Erin K. Box, Christopher A. Cleveland, Ania A. Majewska, Michael J. Yabsley

**Affiliations:** 1 Southeastern Cooperative Wildlife Disease Study, University of Georgia, Athens, Georgia USA; 2 Warnell School of Forestry and Natural Resources, University of Georgia, Athens, Georgia USA; 3 Odum School of Ecology, University of Georgia, Athens, Georgia USA; 4 Emory University, Biology Department, Atlanta, Georgia USA

**Keywords:** Behavioural ecology, Ecological epidemiology

## Abstract

*Dracunculus medinensis*, the causative agent of Guinea worm disease in humans, is being reported with increasing frequency in dogs. However, the route(s) of transmission to dogs is still poorly understood. Classical transmission to humans occurs via drinking water that contains cyclopoid copepods infected with third stage larvae of *D. medinensis*, but due to the method of dog drinking (lapping) compared to humans (suction and/or retrieval of water into containers), it seems unlikely that dogs would ingest copepods readily through drinking. We exposed lab raised beagles to varying densities of uninfected copepods in 2 liters of water to evaluate the number of copepods ingested during a drinking event. We confirmed dogs can ingest copepod intermediate hosts while drinking; however, low numbers were ingested at the densities that are typically observed in Chad suggesting this transmission route may be unlikely. Overall, the relative importance of the classic transmission route and alternate transmission routes, such as paratenic and transport hosts, needs investigation in order to further clarify the epidemiology of guinea worm infections in dogs.

## Introduction

Guinea worm disease (GWD), caused by *Dracunculus medinensis*, is a painful and debilitating disease that is historically widespread in humans among sub-Saharan Africa and southern Asia. The international campaign to eradicate GWD has been extremely successful and has resulted in a decrease of >3.5 million human cases annually in 21 countries to only 49 human cases in four countries in 2019 (43 in Chad, 4 in South Sudan, 1 in Angola, 1 in Cameroon); however, infections of animals occur in four countries (Chad, Ethiopia, Mali, and Angola)^1^. The vast majority of these animal infections are from Chad (n = 1,901) and in domestic dogs in Chad (n = 1,855)^1^. In Chad, there are limited numbers of human infections despite the significant increase in the number of infected domestic dogs and domestic cats, suggesting alternative transmission pathways may be involved in some animal infections^2,3^. Recent data indicates that amphibians can be paratenic hosts and fish can be transport hosts, possibly contributing to this unusual epidemiology^4–6^. Two recent genomic studies showed that the *D. medinensis* derived from humans and animals are the same species and part of the same population of worms^7,8^. Thus, animal infections are a major concern for the eradication campaign as they can contaminate water sources, which thereby could increase the risk of human or additional animal infections^2^.

In humans, transmission of *D. medinensis* is considered to occur through the ingestion of infected copepods in contaminated drinking water. Effective control strategies were developed and implemented to break this transmission route to humans. However, the standard control strategies used for humans (e.g., filtering water, using safe water sources) cannot easily be applied to animal populations. Currently, given the unusual epidemiology compared to typical human infections, there is debate on the source of dog infections^2,3,9^. They can become infected through ingestion of infected copepods but could also become infected through ingestion of infected amphibian paratenic or fish transport hosts. In a first step to investigating the relative importance of these different transmission routes, we conducted this study to determine if dogs ingest copepods during a drinking event and, if so, to quantify ingestion at different copepod densities. When dogs lap, liquid adheres to the tongue and is ingested against gravity as opposed to suction drinking, the creation of a vacuum to ingest water, used by humans or some animals (e.g., baboons); therefore, dogs may not readily ingest copepods^10^.

## Methods

To examine whether dogs ingest copepods during a drinking event, four lab-raised beagles (Ridglan Farms, Inc.) of the same age were individually housed in a climate-controlled (70 °F, 21 °C) facility and provided food and water ad libitum (except for the 12 hrs before being provided copepod-spiked water samples wherein water was withheld, but food was made available). Lapping trial containers consisted of a 2.5 L square glass container (Pyrex®) attached to a metal 2-in deep catch basin with Velcro®. The catch basin caught any water or copepods that were splashed out but not consumed during a drinking event. Lapping trial containers were secured to the wall of the dog enclosure to reduce splashing and spilling.

Dogs were fasted from water 12 hrs prior to each trial. During each drinking event, dogs were presented with two liters of dechlorinated water spiked with one of five copepod density groups (0, 50 [25/L], 100 [50/L], 500 [250/L], and 1000 [500/L]) (Table 1). Copepods were lab-raised from an initial group of copepods collected from Bishop, GA, USA, and housed at the Aquatic Biotechnology and Environmental Lab at the University of Georgia, Athens, GA, USA. Copepods were identified as a *Macrocyclops* sp. through morphology and analysis of partial cytochrome oxidase subunit 1 (COI) gene sequence as described^11^. In Africa, species of *Mesocyclops*, *Thermocyclops*, and *Macrocyclops* are believed to be the most important intermediate hosts for *D. medinensis*^12^.

**Table 1:** Design of the dog drinking trial for a single replicate.

	Dog ID	No. copepods/2L of water				
		Week 1	Week 2	Week 3	Week 4	Week 5
Tuesday	Dog 1	0	1000	500	50	100
	Dog 2	1000	0	50	100	500
Thursday	Dog 3	50	100	500	1000	0
	Dog 4	100	500	50	0	1000

Each dog was exposed to the water within their regular enclosures for one hour to allow for natural lapping conditions. Experimenters left the room during this period to avoid distracting the dogs. After an hour, water was removed, and the catch basin rinsed to recover any copepods that may have adhered to the basin wall. Remaining copepods were quantified and the amount of water consumed was recorded. We ran three replicates of the drinking trials, yielding a total of 60 observations. Each dog was exposed to one of the five different copepod concentrations weekly for five weeks. All experimental methods were reviewed and approved by the University of Georgia’s Institutional Animal Care and Use Committee (A2016 11-004) and were performed in accordance with relevant guidelines and regulations.

We tested whether the number of consumed copepods was influenced by initial copepod concentration. We fit a generalized linear mixed effects model (GLMM; lme4 package^13^) with negative binomial errors. The initial concentration of copepods was the explanatory variable and the offset was volume of water consumed to account for variation in the amount of water ingested by each dog. To account for repeated measures of each dog, we included dog identification and replicate number as random effects. We used R programming software for the statistical analyses^14^.

### Ethical approval and informed consent

All experimental methods were reviewed and approved by University of Georgia’s Institutional Animal Care and Use Committee (A2016 11-004) and in accordance to relevant guidelines and regulations.

## Results

**Figure 1:**
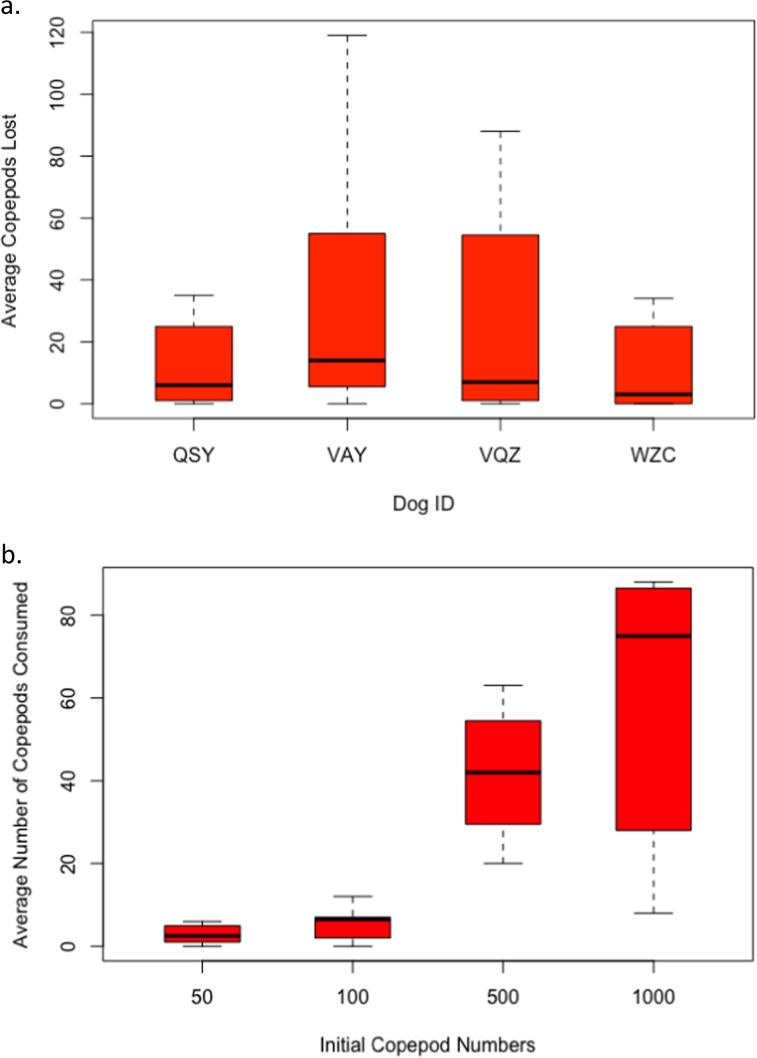
(**a**) Average number of copepods consumed during a drinking event by each individual dog. (**b**) Average number of copepods consumed during a drinking event at each copepod density presented to the dogs. The black bars in the figures represent the means.

Copepod consumption varied somewhat among individuals (Fig. 1a), and statistical analysis showed that the number of copepods consumed was positively influenced by the initial number of copepods presented to a dog (*z* = 6.6, p < 0.001). The average number of copepods consumed per copepod concentration were: 3 copepods for the 50 copepods group, 7 for the 100 group, 41 for the 500 group, and 96 for the 1000 group (Fig. 1b).

## Discussion

Our data indicate that under experimental conditions, dogs may ingest copepods during a drinking event. Thus, it is theoretically possible that enough infected copepods could be ingested to result in dog infections if the copepod infection rate was high enough. We also found that higher densities of copepods led to higher ingestion rates. In Chad, copepod density varies seasonally and between water-bodies. A recent survey found that the average annual number of copepods in four ponds in Chad was 100–200 copepods/L with periodic and short-term spikes of 400–700 copepods/L, primarily in the summer months (T. Moundai, unpublished data); however, additional variation may be present in other regions of Chad. Our highest density tested (1000 [500 copepods/L]) represents the highest naturally observed densities in Chad, while the lower and middle doses (100 [50/L] and 500 [250/L]) represent more frequently detected densities. If numbers of copepods ingested under natural conditions is similar to our experimental data, numerous drinking events from contaminated water bodies may be necessary for *Dracunculus* transmission to occur. It is important to note the prevalence of infected copepods in a contaminated pond is poorly understood but is presumed to be relatively low based on past studies (range of 0.5–33.3%, average of 5.2%)^15–17^. Also, because both a female and male worm are required to complete the life cycle, and copepods infected with more than one *Dracunculus* L3 are likely to die^18^, multiple infected copepods must be ingested. Although it is theoretically possible to have a single male and single female larvae mature, numerous experimental studies have shown that many ingested larvae fail to mature in definitive hosts; thus multiple infected copepods likely have to be ingested^4,15^. At copepod densities of 50 [25/L] and 100 [50/L], 3–7 copepods were consumed on average (Fig. 1b); thus the likelihood of a dog ingesting multiple infected copepods during the two to three week time period in which the copepods are infected with mature L3’s, and thus infections to a definitive host may be low. However, it cannot be ruled out or minimized, especially within the context of eradication.

Other considerations must be addressed during future studies, ideally under field conditions. Our dogs were maintained indoors under climate-controlled conditions (21 °C), whereas there are more extreme temperatures (40 °C to 43 °C) during the peak transmission season in Chad. To partially account for this, water was withheld from dogs for 12 hrs (maximum allowed). Also, our copepods were collected from Georgia, USA which may behave differently than Chadian copepods. Relatively small glass dishes were used in this study; however, wild copepods likely have more opportunities to flee from dogs within larger water volumes. Finally, under natural conditions, copepods may flee into substrate of water-bodies, which is especially known to occur during daylight hours^19^. Another important consideration is that dogs in Chad will often wade and lie in water-bodies leading to disruption of copepods, which could influence the likelihood of ingestion. It is possible that this disruption could decrease risk of ingestion as copepods flee the disturbance. This is assuming that the behavior of infected copepods is similar to uninfected copepods. One study by Onabamiro (1954) found that infected copepods were more sluggish than uninfected copepods; however, a large number of parasites were provided to copepods so many likely had multiple larvae which could have impacted their behavior more than what typically occurs in nature (copepods infected with a single larva)^16,20^.

In conclusion, despite our small sample size, our data indicate that dogs can ingest relatively few copepods while drinking, but there are still many factors to investigate to determine the primary transmission route(s) of *D. medinensis* in the remaining GWD-endemic countries. Other possibilities include ingestion of amphibian paratenic hosts or fish transport hosts^2,4–6,15^. Currently, there are many interventions in place to minimize transmission risk including: tethering of infected dogs, treatment of potentially contaminated water bodies with Abate®, and the burning or burial of fish entrails, but dog infections continue to occur suggesting that improved adherence to interventions or new interventions are necessary to interrupt transmission. Continued evaluation of transmission routes, in the field and theoretically, may help refine or develop interventions.

**Disclaimers**. The opinions expressed by authors contributing to this journal do not necessarily reflect the opinions of the institutions with which the authors are affiliated.

## Data Availability

The datasets generated during this study are available from the corresponding author upon reasonable request.
